# Supplementary material for: Patterns of Intron Gain and Loss in Fungi
Source: PLoS Biol. 2004 Nov 30;2(12):e422. doi: 10.1371/journal.pbio.0020422 (PMC532390; doi:10.1371/journal.pbio.0020422)
Supplement: Table S1 — Also available at http://genes.mit.edu/NielsenEtAl/. (4.3 MB ZIP). [file pbio.0020422.st001.zip › NielsenEtAl/html/391.html]

AN1883.1.NCU02639.1.MG07169.1.FG06098.1


```
 CLUSTAL W (1.82) Multiple Sequence Alignments - Introns Inserted


Sequence 1: NCU02639.1	408 aa
Sequence 2: MG07169.1	406 aa
Sequence 3: FG06098.1	415 aa
Sequence 4: AN1883.1	468 aa
Alignment Length: 470 aa
Number Identitical Residues: 216 aa
Alignment Score (without introns) 11181


MG07169.1 	--MSKGRVCL2AYSGG1LDTS0---~------GYTVV~CFL~ANVGQEE~DWDEVKAKAK
NCU02639.1	MSESKGRVCL2AYSGG1LDTS0---~------GYTVV~CFL~ANVGQEE~NWDEVRAKAK
FG06098.1 	--MSKGRVCL2AYSGG1LDTS~TIL~KWLILEGYEVV0CFL~ADVGQVE~DFDAVEKKAL
AN1883.1  	--MGKGKICV~AFSGG~LDTS~VIL1KWLIDEGYEVV~AFT1ADVGQEE1DFAAIKEKAL
          	   .**::*: *:*** ****     .    .** ** .*  *:*** * ::  :. ** 

MG07169.1 	KLGAEEMVILDLQK~EFVEQIVFRAIQCNAIYEDRYLLGTSLARPIIARAQVRVAE~QYN
NCU02639.1	KLGAEEMVILDLQQ~EFVDQIVFRAIQCNAIYEDRYLLGTSLARPIIARAQVRVAE~QYN
FG06098.1 	ALGAERMIIENLQK~EFIEQIVFRAIQCNAIYEDRYLLGTALARPVIARAQVRVAE~KYN
AN1883.1  	KLGAVKAEVVDLRR1EFVEELCFPAIACNAIYENVYLLGTSLARPVIARAQIEVAK0REG
          	 *** .  : :*:: **:::: * ** ******: *****:****:*****:.**: : .

MG07169.1 	CQYVSHGCTGKG~ND~QVRFELAFK~ACNPSIEVIAPWRLPEFISKFQGRQDLLKYAEEK
NCU02639.1	CEFVSHGCTGKG~ND~QVRFELAFK2ACNPEIKVIAPWRLPEFCARFQGRQDLLKYAADK
FG06098.1 	CNLLSHGCTGKG0ND2QVRFELAFK~ACNPSIKIIAPWRLPEFCEKFQGRQDLLKFAAEN
AN1883.1  	CFAVSHGCTGKG~ND~QVRFELAFY~ALQPDIKVIAPWRDPRFYERFAGRNDLLAYAAEK
          	*  :******** ** ********  * :*.*::***** *.*  :* **:*** :* ::

MG07169.1 	NIPVSSSPKAPWSMDDNLVHCSYEAGVLEDPDHTPPKELWTRTVDPVDAPATPQEFTVEF
NCU02639.1	NIPVSSTPKAPWSMDDNLVHCSYEAGILEDPNHTPPKELWTRTVDPQDAPNTPQEFTIVF
FG06098.1 	NIPVSSTPKAPWSQDENLVHCSYEAGILEDPDHTPPKDLWTQTVDPLEAPDKPFNFAVHF
AN1883.1  	GIPVTSTKAKPWSMDENLAHCSYEAGILEDPDITPPTDMWKLTVDPLAAPDKPEDFTVHF
          	.***:*:   *** *:**.*******:****: ***.::*. ****  ** .* :*:: *

MG07169.1 	EKGIPTKVTVDG----KETTGSVEVFKLLNKLGHDHGVGRIDI~VENRFIGLKSRGCYDT
NCU02639.1	EKGIPVKVIVDG----KETTGSVELFKLLNKIGHDHGVGRVDI~VENRFIGLKSRGCYDT
FG06098.1 	EKGLPVKVATPD----QEATDSVELFKLLNKIGHDHGVGRIDI2VENRWIGLKSRGCYDT
AN1883.1  	EKGLPVKLEYTENGQQKTATDAVDIFLTANAIARRNGIGRIDI0VENRFIGIKSRGCYET
          	***:*.*:    ....: :*.:*::*   * :.: :*:**:** ****:**:******:*

MG07169.1 	PGLTIARLAHL~DLE~GLVMDSKVREIRDQFCTLSWSR~QLYNGMYFSPEREF2VENSII
NCU02639.1	PGLTIARLAHL~DLE1GLVMDSRVRELRDQFVTISWSR~QLYNGMYFSPEREF2VENSII
FG06098.1 	HGLTIARLAHV~DLE~GLVLDSKVRKLRDQFVTVEWSQ~CLYSGMYFSPEREW~LDEAIV
AN1883.1  	PGLTCLRSAHV2DLE~GLVLDREVRALRDQFVTINYSK0LLYNGLYFSPEREF~LEQAIP
          	 ***  * **: *** ***:* .** :**** *:.:*:  **.*:*******: ::::* 

MG07169.1 	FSQENVTGQVRLAVYKGNTYVLGRKSDASNLYSEQDASMDSLEGFSPMDTTGFIEINAIR
NCU02639.1	FSQQNVTGEVRMMAYKGNAYVLGRKSDASNLYSEADASMDSLEGFSPMDTTGFIEINAIR
FG06098.1 	SAQKGVNGQVRLQVYKGNVYVLGRSSETSSLYSQEDASMDSLDTFSPMDSTGFIAIQSIR
AN1883.1  	ASQKSVNGKVRCRAYKGNMIILGRSSETEKLYDMSESSMDEIGDFAPTETTGFIGVSAIR
          	 :*:.*.*:**  .****  :***.*::..**.  ::***.:  *:* ::**** :.:**

MG07169.1 	LKKYGLQKIKDGKPL~TQS------------------------------~----------
NCU02639.1	LKKYGLQKIKDGKPL~TQS------------------------------~----------
FG06098.1 	LEKYGVQKAKDGQPL~SQS------------------------------~----------
AN1883.1  	LKKYGQMKQAAGEKL2KVIWNNTLEGYPATSAPVSLRIQGGQAWSETHL2SNASSDENPA
          	*:***  *   *: * .   ..: .. .::::. :   ....: :.:   :.:::....:

MG07169.1 	---------
NCU02639.1	---------
FG06098.1 	---------
AN1883.1  	DYGPNPISD
          	. .... :.
```
